# Horizontal Inequity in the Utilization of Maternal and Reproductive Health Services: Evidence From the 2018 Nigeria Demographic and Health Survey

**DOI:** 10.3389/frhs.2022.791695

**Published:** 2022-04-13

**Authors:** Bolaji Samson Aregbeshola, Olanrewaju Olaniyan

**Affiliations:** ^1^School of Medicine and Public Health, College of Health, Medicine and Wellbeing, The University of Newcastle, Callaghan, NSW, Australia; ^2^Department of Economics, University of Ibadan, Ibadan, Nigeria

**Keywords:** horizontal equity, health inequities, utilization of health services, maternal health, reproductive health, Nigeria

## Abstract

**Background:**

Maternal mortality and poor reproductive health outcomes remain major public health challenges in low-resource countries. The Sustainable Development Goals have proposed new targets to reduce global maternal mortality ratio to 70 per 100,000 live births and ensure universal access to sexual and reproductive healthcare services by 2030. Inequity in the utilization of maternal and reproductive health services leads to poor reproductive health outcomes and maternal mortality. Despite reduction in global maternal mortality over the decades, the level of maternal mortality remains unacceptably high in Nigeria with limited attention given by governments to addressing health inequities. This study aimed to examine horizontal inequity in the utilization of maternal and reproductive health services in Nigeria.

**Methods:**

Secondary data from the 2018 Nigeria Demographic and Health Survey were utilized to examine horizontal inequity in the utilization of maternal and reproductive health services such as postnatal care, delivery by cesarean section, modern contraceptive use, and met need for family planning. Equity was measured using equity gaps, equity ratios, concentration curves, and concentration indices. All analyses were performed using ADePT 6.0 and STATA version 14.2 software.

**Results:**

The overall coverage level of postnatal care, delivery by cesarean section, modern contraceptive use, and met need for family planning was 20.81, 2.97, 10.23, and 84.22%, respectively. There is inequity in the utilization of postnatal care, delivery by cesarean section, and modern contraceptive favoring the rich, educated, and urban populations. Met need for family planning was found to be almost perfectly equitable.

**Conclusion:**

There is inequity in the utilization of maternal and reproductive health services in Nigeria. Inequity in the utilization of maternal and reproductive health services is driven by socioeconomic status, education, and location. Therefore, governments and policymakers should give due attention to addressing inequities in the utilization of maternal and reproductive health services by economically empowering women, improving their level of education, and designing rural health interventions. Addressing inequities in the utilization of maternal and reproductive health services would also be important toward achieving the Sustainable Development Goal targets 3.1 and 3.7.

## Introduction

Maternal mortality and poor reproductive health remain major challenges in low-resource countries. About 300,000 women died around the world in 2017, yielding an overall maternal mortality ratio of 211 per 100,000 live births (1). Sub-Saharan Africa (SSA) accounts for 66% (196,000) of all maternal deaths per year worldwide in 2017, yielding a maternal mortality ratio of 542 per 100,000 live births (1). Although there has been reduction in global maternal mortality over the decades, the level of maternal mortality remains unacceptably high in Nigeria with a maternal mortality ratio at 917 per 100,000 live births (2). Consequently, Nigeria contributes 23% of global maternal deaths (1). Nigeria failed to achieve Millennium Development Goal (MDG) 5, which aimed to reduce maternal mortality ratio by three quarters between 1990 and 2015 (3). The country was also off track in achieving universal access to reproductive health. Giving birth in Nigeria is a dance with death for mothers (4).

Inequity in the utilization of health and health outcomes remains a major challenge in low- and middle-income countries (LMICs) (5). Inequity in the utilization of maternal and reproductive health services leads to poor reproductive health outcomes and maternal mortality. Inequity in the utilization of healthcare services remains a policy-relevant issue in both developed and developing countries (5). There is no consensus among scholars on the definition of equity (6, 7). Equity, in some studies, refers to the distribution of resources, benefits, and welfare among different sub-groups of a population based on need, taking into account issues of fairness and justice (6, 8). It is also concerned with equal access to health services, equal health status for all, and utilization of healthcare based on needs (8, 9). Inequity in healthcare is the unfair or unjust distribution and utilization of healthcare resources based on income or socioeconomic status (SES) as well as demographic or other factors but not based on need (8–10). Equity in healthcare is also categorized into horizontal and vertical equity (8, 11). While horizontal equity means that individuals in equal need of healthcare should receive the same treatment irrespective of age, SES, and race (11–13), vertical equity means that individuals with unequal healthcare need should receive unequal treatment of healthcare (11, 13). Equity in healthcare is an overarching goal of many healthcare systems around the world (14).

In 2015, universal health coverage (UHC) was included as a goal of Sustainable Development Goals (SDGs) (15). UHC aims to increase equity in access to quality healthcare services and reduce associated financial risk (16). The SDGs have proposed new targets to reduce global maternal mortality ratio (MMR) to 70 per 100,000 live births and ensure universal access to sexual and reproductive healthcare services by 2030 (15). However, there exist large inequities in the utilization of maternal and reproductive health services within and between countries, and across socioeconomic groups (17–19). Tracking progress toward narrowing the gap in the utilization of maternal and reproductive health services among the poor and better-off households is critical toward achieving the SDGs. Therefore, there is a need for further studies to understand inequity in the utilization of maternal and reproductive health services. Ensuring improvement in health equity is as important as improving maternal and reproductive health.

Some empirical studies have been conducted on equity in the utilization of maternal and reproductive health services in LMICs (18, 20–32). Results from previous studies show that there are stark disparities in the utilization of maternal and reproductive health services. However, there is limited evidence on horizontal inequity in the utilization of maternal and reproductive health services in Nigeria focusing on use of postnatal care (PNC), delivery by cesarean section, modern contraceptive use, and met need for family planning and using the most recent nationally representative household survey. Furthermore, evidence regarding equity in the utilization of reproductive health services is mixed with very limited studies on equity in the utilization of PNC and delivery by cesarean section (19). For instance, Adetsav examined equity gap in the utilization of maternal health services such antenatal care (ANC), skilled birth attendants (SBA), and health facility-based delivery (HFBD) using binary logistic regression and two data sets [2008 and 2013 Nigeria Demographic and Health Surveys (NDHS)] (33). Results showed that there were wide differences in equity gap in SBA and delivery in health facility for rural–urban and pro-rich gaps but ANC visit improved greatly with the richest wealth quintile being favored (33). Using four rounds of the NDHS (1999, 2003, 2008, and 2013) and concentration index, Nghargbu and Olaniyan examined inequity in maternal and child healthcare utilization focusing on ANC, delivery using SBA, and child immunization (11). The study found that there was a pro-rich inequity in the utilization of ANC, SBA, and child immunization from 1999 to 2013 (11). Nwosu and Ataguba assessed socioeconomic inequalities in ANC utilization and the determinants of these inequalities using the 2013 NDHS (34). They found that no ANC visit was disproportionately concentrated among the poor while at least four ANC visits and a higher number of ANC visits favor the rich (34). Using four rounds of the NDHS (2003, 2008, 2013, and 2018), Theil index, and concentration index, Okoli et al. examined the geographical and socioeconomic inequalities in maternal healthcare utilization in Nigeria (35). It was observed that the utilization of maternal healthcare is lower among poorer and less educated women as well as those living in rural areas and North West and North East geopolitical zones (35). These empirical studies did not focus on use of PNC, delivery by cesarean section, modern contraceptive use, and met need for family planning.

Furthermore, the review of methodological literature revealed that multivariate logistic regression, chi-square test, rate ratios, equity ratios, slope/relative index of inequality, quintile ratios, difference-in-differences method, concentration index, and concentration curve approach were the methods used in evaluating equity in the utilization of maternal and reproductive health services with multivariate logistic regression being the most commonly used method (14, 19). However, concentration curve (CC) and concentration index (CI) are regarded as the best methods in assessing equity in utilization of health services (5, 12). CC provides a complete picture of how the utilization of health services varies across the full distribution of SES while CI quantifies the degree of socioeconomic-related inequality in the utilization of health services (5). Also, previous studies used age, wealth, education, location, ethnicity, religion, and caste as proxies for measuring equity (19, 33).

This study aimed to examine horizontal inequity in the utilization of maternal and reproductive health services in Nigeria. The study adds to the existing literature and contribute to the body of knowledge on equity in the utilization of maternal and reproductive health services by using the 2018 NDHS and focusing on utilization of PNC, delivery by cesarean section, modern contraceptive use, and met need for family planning. The study also contributes to achieving equitable health system in Nigeria by providing insights into the design of a health system that ensures the utilization of maternal and reproductive health services based on need.

## Materials and Methods

### Data Source

Secondary data from the 2018 NDHS were utilized. The 2018 NDHS is a nationally representative survey of men and women aged 15–49 years based on a two-stage sampling technique. The first stage involved the selection of 1,400 Enumeration Areas (EAs) with probability proportional to EA size (the number of households in the EA) (36). A household listing operation was carried out in all selected EAs, and the resulting lists of households served as a sampling frame for the selection of households in the second stage (36). The second stage involved the selection of a fixed number of 30 households in every cluster through equal probability systematic sampling, resulting in a total sample size of ~42,000 households (36). The survey provides updated estimates of basic demographic and health indicators such as fertility, awareness and use of family planning methods, breastfeeding practices, nutritional status of women and children, maternal and child health, adult and childhood mortality, women's empowerment, domestic violence, female genital cutting, prevalence of malaria, awareness and behavior regarding HIV/AIDS and other sexually transmitted infections (STIs), disability, and other health-related issues such as smoking (36). The survey sampled 41,821 women aged 15–49 years. Out of the 42,121 women aged 15–49 years identified in the female survey, 41,821 were successfully interviewed, yielding a response rate of 99% (36). The 2018 NDHS data were collected from August 14, 2018 to December 29, 2018 (36).

### Outcome Variables

The outcome variables were modern contraceptive use (coded as 1 if women used modern contraceptives and 0 otherwise), met need for family planning (coded as 1 if women had met need for family planning and 0 otherwise), delivery by cesarean section (coded as 1 if a woman delivered by cesarean section and 0 otherwise), and use of PNC (coded as 1 if women received a health check within 2 months after delivery and 0 otherwise). In 2013, the World Health Organization (WHO) updated its guidelines on PNC with the recommendation that women and newborns should receive PNC at a health facility for at least 24 h after birth, on day 3 (48–72 h), between days 7 and 14 after birth, and 6 weeks after birth, regardless of the place of delivery (37). The variable for PNC was derived from the question: How long and how often after delivery did the respondent receive health checks?

### Measures of Inequity

Measures of inequity were maternal education, location, and SES. A SES index was constructed using Principal Component Analysis (PCA) based on data from variables on household ownership of assets and housing conditions (38). These variables include ownership of a car/truck, ownership of radio, ownership of refrigerator, ownership of bicycle, ownership of motorcycle, main wall material, main floor material, main roof material, type of fuel for cooking, source of electricity, source of drinking water, time to get to water source, and type of toilet facility used. PCA generated factor score on each household asset. The resulting asset scores were standardized while the standardized scores were used to generate SES quintile as poorest, poorer, middle, richer, and richest.

### Analytical Method

All analyses were performed using STATA version 14.2 software and ADePT version 6.0 developed by the World Bank's Development Research Group (DECRG). Descriptive statistics were used to analyze the demographic and socioeconomic characteristics of the study sample in the form of frequency tables and simple percentages. Utilization of maternal and reproductive health services was compared by education, location, and SES. Equity was measured using equity gaps, equity ratios, concentration curves, and concentration indices. Concentration curve was plotted for each maternal and reproductive health services. Concentration curve and concentration index are regarded as the best methods in assessing equity in healthcare utilization because they are consistent with ranking individuals by wealth rather than health status, they are sensitive to population distribution across socioeconomic groups, and they assess relative inequality rather than absolute inequality (5, 12). CC uses the concept of horizontal equity, i.e., treating people with equal need the same and irrespective of their income. It not only represents overall inequity, but also reflects accurately which social classes are allocated with more resources. “Horizontal” inequity was also represented using CI of need-adjusted use of maternal and reproductive health services. Maternal and reproductive health services were indirectly standardized by age and sex within the sample population to reduce the confounding effects of variables correlated with SES and maternal and reproductive health services (5). The standard error and confidence intervals for each concentration index were also calculated (5). Cluster weights were included in the estimation of CC and CIs. Weighting factors constructed by the Measure DHS were used to adjust for common causes, clustering, and sampling weights.

## Results

### Descriptive Statistics

The characteristics of the study sample are shown in Table 1. More than two-thirds (69.07%) of the study sample were currently married. About 65% of respondents were employed. More than two-thirds (68.88%) of the study sample reported that their partner worked in the informal sector. More than half (59.39%) of the study sample reside in rural areas. Two-thirds (66.36%) of the study sample had five or more members in the household. More than two-thirds (67.55%) of respondents were underweight. Details are given in Table 1.

**Table 1:** Descriptive statistics.

**Variable**	***N*** **= 41,821**	
	***N***	**%**
**Maternal age**		
15–24	15,267	36.51
25–34	13,200	31.56
35+	13,354	31.93
**Maternal education**		
No education	14,398	34.43
Primary education	6,383	15.26
Secondary education	16,698	39.93
Higher education	4,342	10.38
**Partner's education**		
No education	10,196	35.29
Primary education	4,436	15.36
Secondary education	9,769	33.82
Higher education	4,487	15.53
**Marital status**		
Never married	10,669	25.51
Currently married	28,888	69.08
Formally married	2,264	5.41
**Maternal occupation**		
Unemployed	14,766	35.31
Employment	27,055	64.69
**Partner's occupation**		
Not working	1,138	3.94
Formal worker	11,215	38.82
Informal worker	16,535	57.24
Missing	12,933	
**Location**		
Urban	16,984	40.61
Rural	24,837	59.39
**Geo-political zone**		
North Central	7,772	18.58
North East	7,639	18.27
North West	10,129	24.22
South East	5,571	13.32
South South	5,080	12.15
South West	5,630	13.46
**Religion**		
Christianity	20,506	49.04
Islam	20,959	50.12
Traditionalist/other	356	0.85
**Parity**		
<3 children	22,688	54.25
3–4 children	9,464	22.63
5 or more children	9,669	23.12
**Household size**		
<5 members	14,067	33.64
Five or more members	27,754	66.36
**Gender of household head**		
Male	34,614	82.77
Female	7,207	17.23
**Health insurance**		
No	40,704	97.33
Yes	1,117	2.67
**Ethnicity**		
Yoruba	5,372	12.85
Igbo	6,714	16.05
Hausa/Fulani	13,718	32.80
Others	16,017	38.30
**Birth order**		
1	4,649	15.50
2–4	13,252	44.19
5+	12,091	40.31
Missing	11,829	-
**SES**		
Poorest	7,747	18.52
Poorer	8,346	19.96
Middle	8,859	21.18
Richer	8,840	21.14
Richest	8,029	19.20
**Currently pregnant**		
No	37,630	89.98
Yes	4,191	10.02
**BMI**		
Underweight (<18.5 kg/m^2^)	28,250	67.55
Normal (18.5–24.9 kg/m^2^)	9,127	21.82
Overweight (25.0–29.9 kg/m^2^)	2,686	6.42
Obese (30 kg/m^2^ or higher)	1,758	4.20

### Inequity in the Utilization of Maternal and Reproductive Health Services

Table 2 present results of utilization of maternal and reproductive health services by SES quintiles.

**Table 2:** Utilization of maternal and reproductive health service by SES.

**Utilization of maternal and reproductive health services**	**Q1 (%)**	**Q2 (%)**	**Q3 (%)**	**Q4 (%)**	**Q5 (%)**	**Total (%)**	**Equity gap (Q5–Q1) (%)**	**Equity ratio (Q5/Q1)**	***p*** **-value**
Postnatal care checkup	13.00	15.35	22.35	27.87	30.21	20.81	17.21	2.32	<0.001^*^
Delivery by cesarean section	0.32	0.86	2.05	3.93	10.37	2.97	10.05	32.41	<0.001^*^
Modern contraceptive use	4.90	7.71	12.39	18.17	24.64	12.50	19.74	5.03	<0.001^*^
Met need for family planning	78.99	77.06	73.35	71.93	75.67	75.57	−3.42	0.96	<0.001^*^

* *p < 0.05 = statistically significant*.

The overall coverage level of postnatal care, delivery by cesarean section, modern contraceptive use, and met need for family planning was 20.81, 2.97, 10.23, and 84.22%, respectively. The utilization of PNC is higher among women from the richest quintile compared to those from the poorest quintile, and this was statistically significant. The rate of delivery by cesarean section is higher among women from the richest quintile compared to those from the poorest quintile, and this was statistically significant. The utilization of modern contraceptive among women from the richest quintile is five times more than those from the poorest quintile, and this was statistically significant. Equity ratio for met need for family planning by SES is 0.96. Results of utilization of maternal and reproductive health services by education are shown in Table 3. The utilization of PNC is higher among women with higher education compared to women with no education, and this was statistically significant. The rate of delivery by cesarean section is higher among women with higher education compared to women with no education, and this was statistically significant. The utilization of modern contraceptive among women with higher education is five times more than those with no education and this was statistically significant. Met need for family planning is higher among women with higher education compared to women with no education, and this was statistically significant. Table 4 presents results of utilization of maternal and reproductive health services by location. The utilization of PNC is higher among women living in urban areas compared to those living in rural areas, and this was statistically significant. The rate of delivery by cesarean section is higher among women living in rural areas compared to those living in rural areas, and this was statistically significant. The utilization of modern contraceptive among women living in urban areas is 1.95 times more than those living in rural areas, and this was statistically significant. Equity ratio for met need for family planning by location is 0.98.

**Table 3:** Utilization of maternal and reproductive health service use by education.

**Utilization of maternal and reproductive health services**	**No education (%)**	**Primary education (%)**	**Secondary education (%)**	**Higher education (%)**	**Total (%)**	**Equity gap (%)**	**Equity ratio**	***p*** **-value**
Postnatal care checkup	11.38	23.87	29.27	31.84	20.81	20.46	2.80	<0.001^*^
Delivery by cesarean section	0.60	1.76	4.13	13.29	2.97	12.69	22.15	<0.001^*^
Modern contraceptive use	4.83	14.19	18.84	25.13	12.50	20.3	5.20	<0.001^*^
Met need for family planning	77.87	71.96	73.50	78.34	75.57	0.47	1.01	<0.001^*^

* *p < 0.05 = statistically significant*.

**Table 4:** Utilization of maternal and reproductive health service use by location.

**Utilization of maternal and reproductive health services**	**Rural (%)**	**Urban (%)**	**Total (%)**	**Equity gap (%)**	**Equity ratio**	***p*** **-value**
Postnatal care checkup	16.39	28.89	20.81	12.50	1.76	<0.001^*^
Delivery by cesarean section	1.46	5.73	2.97	4.27	3.92	<0.001^*^
Modern contraceptive use	9.36	18.25	12.50	8.89	1.95	<0.001^*^
Met need for family planning	76.13	74.54	75.57	−1.59	0.98	0.001^*^

* *p < 0.05 = statistically significant*.

**Figure 1:**
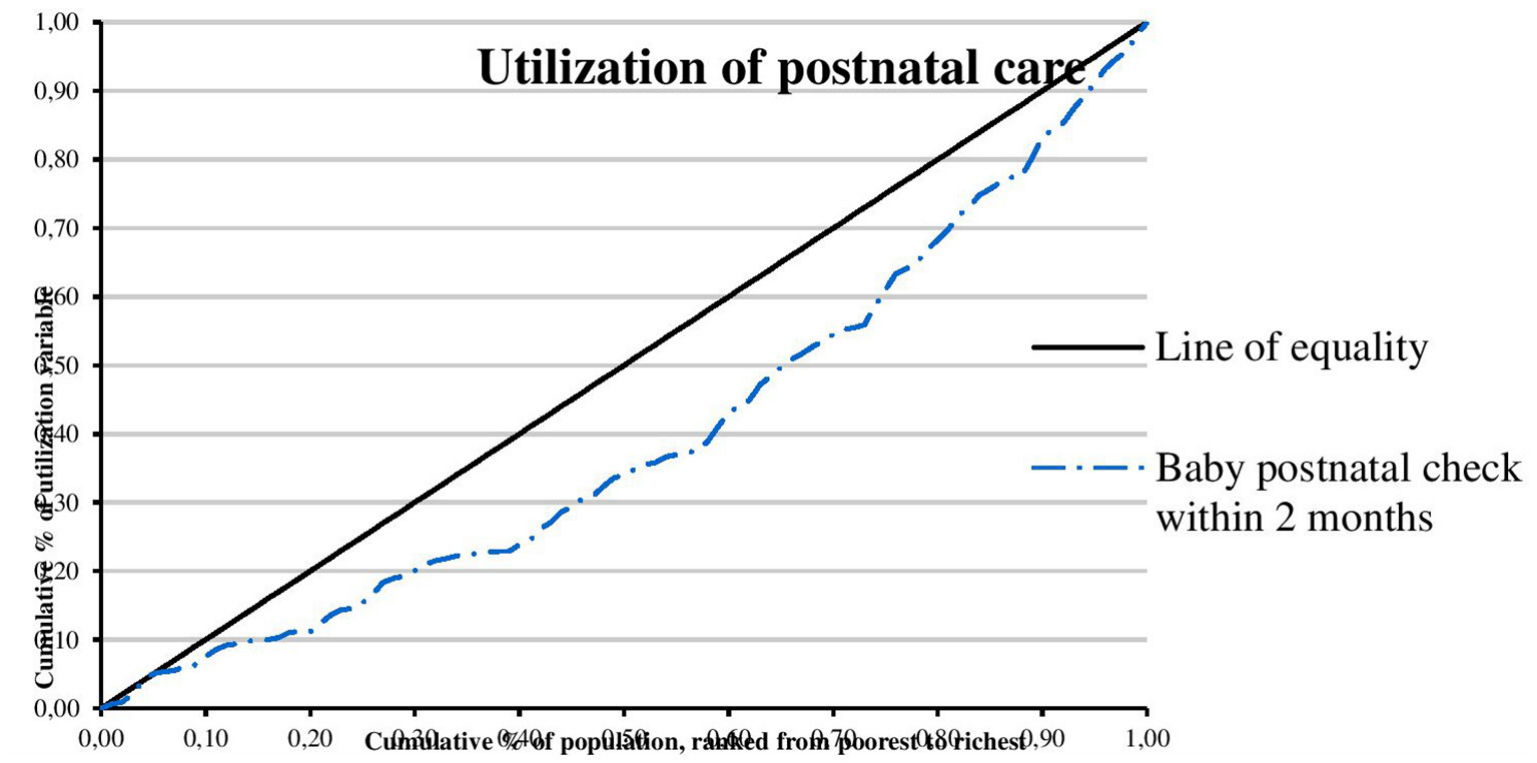
Concentration curve of utilization of postnatal care.

**Figure 2:**
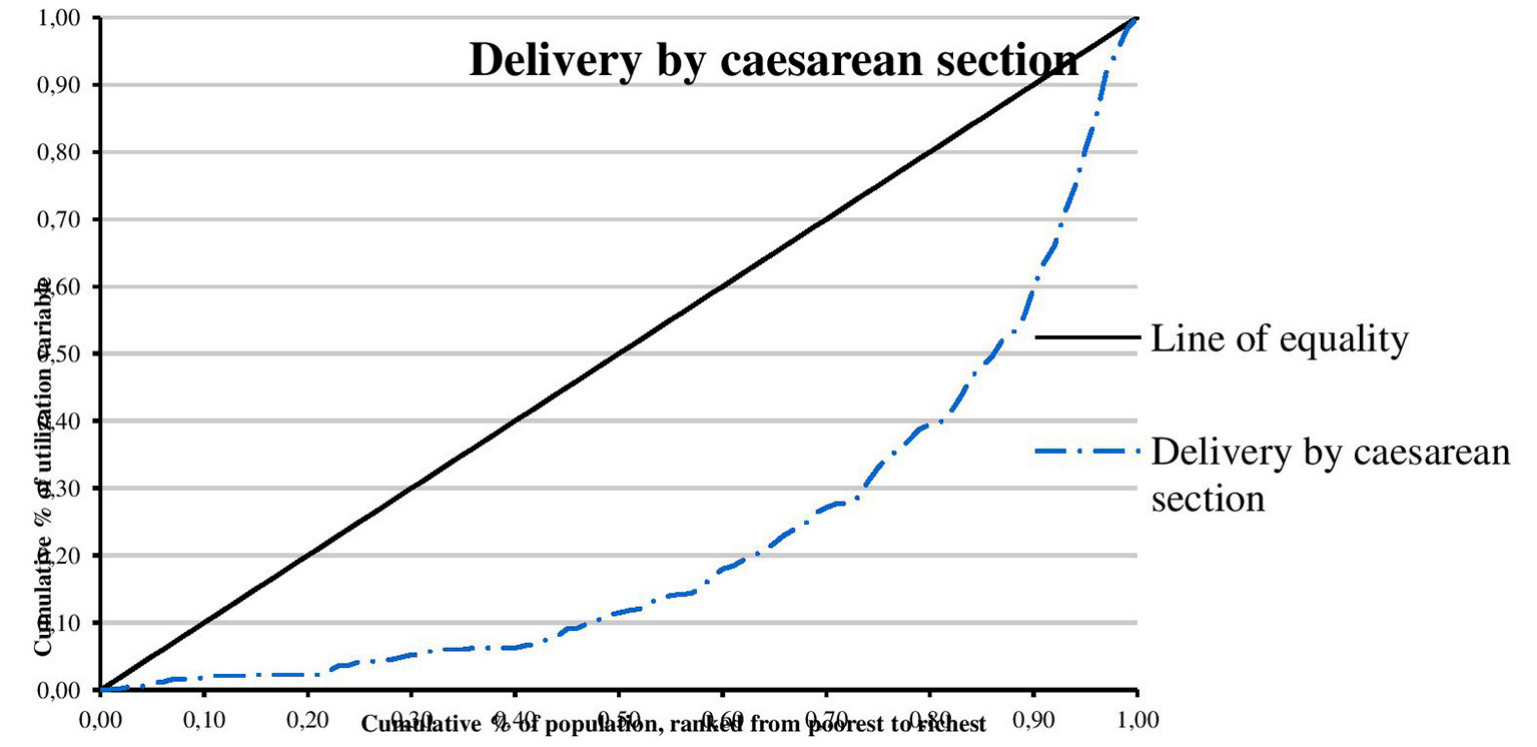
Concentration curve of delivery by cesarean section.

**Figure 3:**
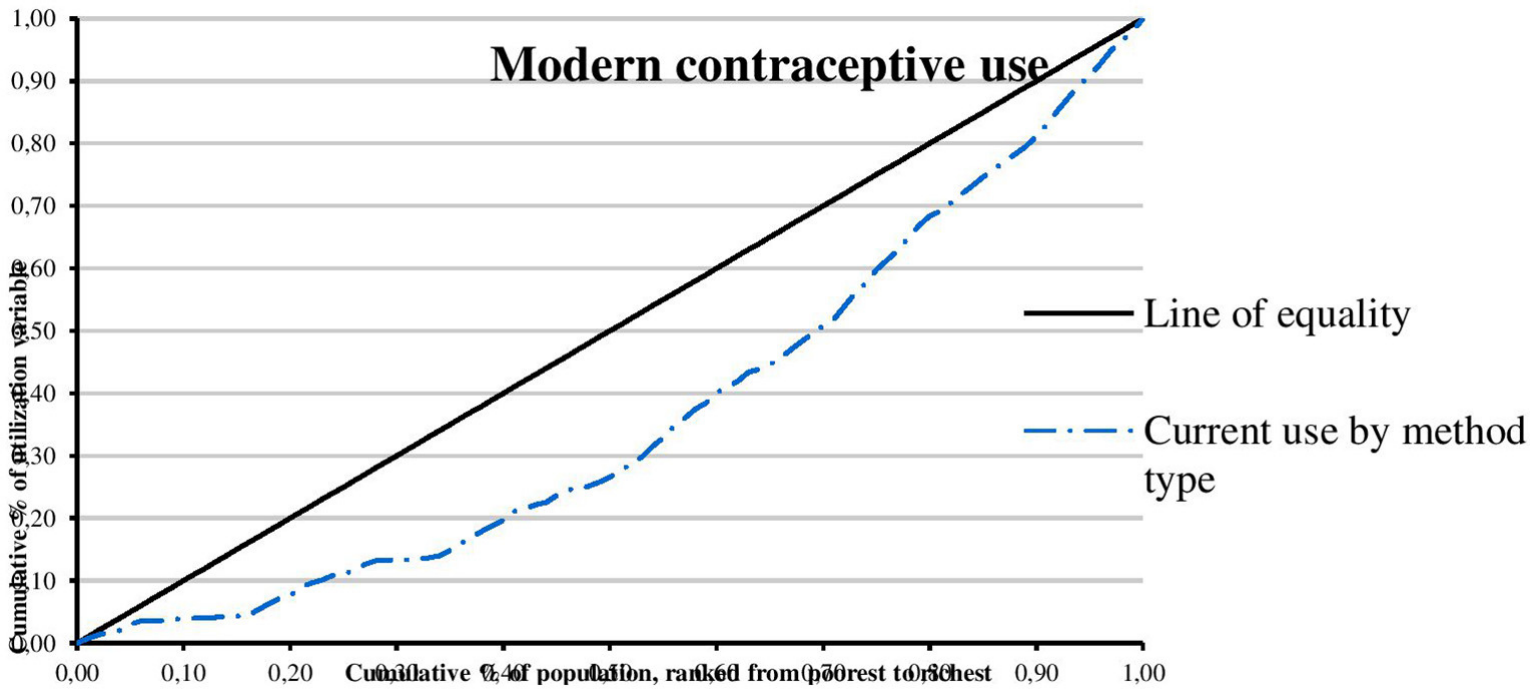
Concentration curve of modern contraceptive use.

**Figure 4:**
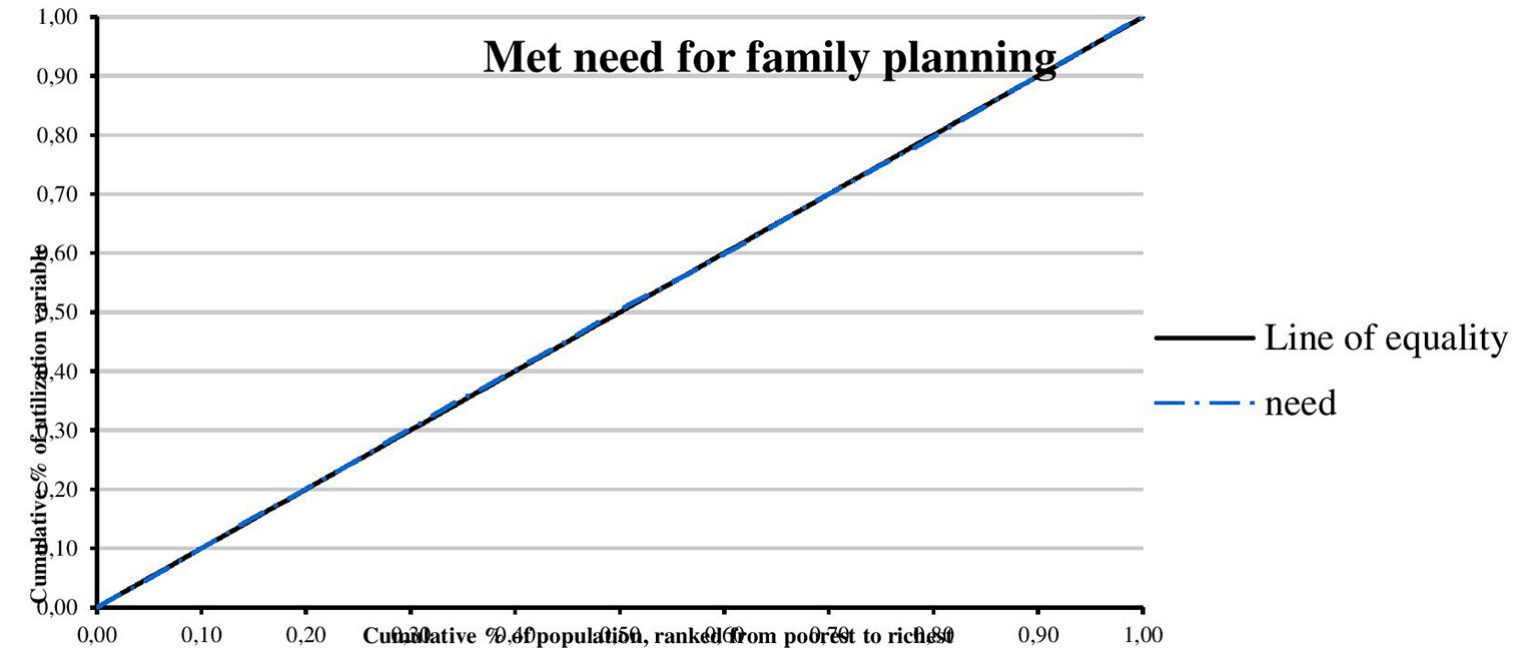
Concentration curve of met need for family planning.

The concentration curves of utilization of maternal and reproductive health services are shown in Figures 1–4. Results show that there is inequity in the utilization of PNC, delivery by cesarean section, and modern contraceptive favoring the rich. Met need for family planning was almost perfectly equitable across socioeconomic quintiles. The standardized concentration index was 0.352, 0.736, 0.471, and 0.035 for utilization of PNC, delivery by cesarean section, modern contraceptive use, and met need for family planning, respectively (see Table 5).

**Table 5:** Horizontal inequity in the utilization of maternal and reproductive health service.

**Maternal and reproductive health service use**	**CI**	**SE**	**95% Confidence Intervals**
Postnatal care checkup	0.352	0.01	0.319–0.386
Delivery by cesarean section	0.736	0.02	0.705–0.768
Modern contraceptive use	0.471	0.01	0.437–0.504
Met need for family planning	0.035	0.00	0.005–0.066

*CI is concentration index; SE, standard error*.

## Discussion

This study examined horizontal inequity in the utilization of maternal and reproductive health services in Nigeria. The study contributes to achieving equitable health system in Nigeria by informing policy decision-making toward improvement in coverage of maternal and reproductive health services for poor women who have greater need for care. The overall coverage levels of PNC, delivery by cesarean section, modern contraceptive use, and met need for family planning were 20.81, 2.97, 10.23, and 84.22%, respectively. Results show that there is inequity in the utilization of PNC, delivery by cesarean section, and modern contraceptive favoring the rich. A possible explanation is that there is a high level of poverty in Nigeria with low purchasing power. This finding is consistent with results from previous studies that show socioeconomic inequities in the utilization of PNC (20, 27, 30, 39), delivery by cesarean section (22–24, 26, 29–32), and modern contraceptive use (21, 28, 40). Consistent with results from a similar study, the study revealed that met need for family planning was almost perfectly equitable across socioeconomic quintiles (25). Met need for family planning refers to a situation where women who want to reduce or delay childbearing are using contraception while unmet need for family planning refers to the condition where women want to avoid or postpone childbearing but are not using any method of contraception (41). Unmet need for family planning could also be described as the discrepancy between fertility preferences of women and use of contraception (41). However, unmet need for family planning is 22% among currently married women (36). The study also found that there is inequity in utilization of maternal and reproductive health services by education. Women with higher education utilize PNC, delivery by cesarean section, and modern contraceptive, and have met need for family planning more than women who had no education. A possible explanation is that there is poor girl child education and lack of knowledge of the advantages of maternal and reproductive health services. This finding is supported by similar studies (20, 23, 30). In this study, there is inequity in utilization of maternal and reproductive health services by location. Women residing in urban areas utilize PNC, delivery by cesarean section, and modern contraceptive more than women residing in rural areas. A possible explanation is that access to maternal and reproductive health services is poor in rural areas. This finding is consistent with results from other studies (23, 30, 42).

### Policy Implication

Findings from this study have implications for policy and the achievement of SDG targets 3.1 and 3.7. The overall coverage levels of PNC, delivery by cesarean section, and modern contraceptive use are low. Governments and policymakers should increase the coverage of maternal and reproductive health services to women of reproductive age. There is inequity in the utilization of PNC, delivery by cesarean section, and modern contraceptive favoring the rich. This implies that interventions aimed at reducing inequity in the utilization of maternal and reproductive health services are not effective. Therefore, there is a need to address the demand-side (lack of health insurance) and supply-side factors (early child marriage, low level of education, low SES, early childbearing, residing in rural areas, ethnicity, high fertility rate, and poor quality of primary healthcare) affecting the utilization of maternal and reproductive health services toward reducing the gap between the poor and the better-off. Despite the implementation of the National Health Insurance Scheme (NHIS) since 2005, health insurance coverage in Nigeria is <5% (43). Out-of-pocket (OOP) payment remains the major source of financing healthcare due to low health insurance coverage (44–48). A recent study shows that the poor bears the burden of OOP payments for healthcare in Nigeria (49). The economic empowerment of women will be critical. Inequity in the utilization of maternal and reproductive health services is also driven by education and location. Governments and policymakers should focus on women who are uneducated and living in rural areas.

### Strength and Limitations of the Study

Findings from this study should be interpreted with caution. First, the study used cross-sectional secondary data rather than longitudinal data. Second, findings from this study are affected by recall bias due to self-reported information. The strength of the study is that the samples were nationally representative and the response rate of the survey interview was high (99%).

## Conclusion

There is inequity in the utilization of maternal and reproductive health services in Nigeria. Inequity in the utilization of maternal and reproductive health services is driven by SES, education, and location. This implies that the Nigerian health system is not performing equitably. Therefore, governments and policymakers should give due attention to addressing inequities in the utilization of maternal and reproductive health services by economically empowering women, improving their level of education, and designing rural health interventions. Addressing inequities in the utilization of maternal and reproductive health services would also be important toward achieving SDG targets 3.1 and 3.7.

## Data Availability Statement

Publicly available datasets were analyzed in this study. This data can be found at: www.dhsprogram.org.

## Ethics Statement

The studies involving human participants were reviewed and approved by in obtaining the micro data, a request was made on the DHS program website on October 14, 2020 and approval was granted to download the data on the same day, hence, there were no ethical issues of concern. The 2018 NDHS was approved by the National Health Research Ethics Committee of Nigeria (NHREC) and the ICF Institutional Review Board. Written informed consent to participate in this study was provided by the participants' legal guardian/next of kin.

## Author Contributions

BSA conceived and designed the study, acquired the data, performed data analysis, interpreted the data, and drafted the manuscript. BSA and OO revised the manuscript for important intellectual content. Both authors have read and approved the final manuscript.

## Conflict of Interest

The authors declare that the research was conducted in the absence of any commercial or financial relationships that could be construed as a potential conflict of interest.

## Publisher's Note

All claims expressed in this article are solely those of the authors and do not necessarily represent those of their affiliated organizations, or those of the publisher, the editors and the reviewers. Any product that may be evaluated in this article, or claim that may be made by its manufacturer, is not guaranteed or endorsed by the publisher.
